# Comparative Analysis of Right Ventricle Fluid Dynamics

**DOI:** 10.3389/fbioe.2021.667408

**Published:** 2021-07-06

**Authors:** Dario Collia, Luigino Zovatto, Giovanni Tonti, Gianni Pedrizzetti

**Affiliations:** ^1^Department of Engineering and Architecture, University of Trieste, Trieste, Italy; ^2^Institute of Cardiology and Center of Excellence on Aging, “G. D'Annunzio” University of Chieti, Chieti, Italy

**Keywords:** cardiac fluid dynamics, right ventricle, left ventricle, biomechanics, numerical simulation, mitral valve, tricuspid valve, mechanical work

## Abstract

The right and left sides of the human heart operate with a common timing and pump the same amount of blood. Therefore, the right ventricle (RV) presents a function that is comparable to the left ventricle (LV) in terms of flow generation; nevertheless, the RV operates against a much lower arterial pressure (afterload) and requires a lower muscular strength. This study compares the fluid dynamics of the normal right and left ventricles to better understand the role of the RV streamlined geometry and provide some physics-based ground for the construction of clinical indicators for the right side. The analysis is performed by image-based direct numerical simulation, using the immersed boundary technique including the simplified models of tricuspid and mitral valves. Results demonstrated that the vortex formation process during early diastole is similar in the two ventricles, then the RV vorticity rapidly dissipates in the subvalvular region while the LV sustains a weak circulatory pattern at the center of the chamber. Afterwards, during the systolic contraction, the RV geometry allows an efficient transfer of mechanical work to the propelled blood; differently from the LV, this work is non-negligible in the global energetic balance. The varying behavior of the RV, from reservoir to conduct, during the different phases of the heartbeat is briefly discussed in conjunction to the development of possible dysfunctions.

## 1. Introduction

The human heart is composed of the right and the left sides that share the same organ and operate in approximate synchrony pumping blood in the pulmonary and systemic circulations, respectively. The two hearts work in parallel and are arranged in series as the pulmonary circulation starts from the right side and ends on the left and vice versa for the systemic circulation, which implies that the amount of blood pumped by the right side is the same pumped by the left. Therefore, the left and the right hearts operate with same timing to generate the same amount of blood flow. Nevertheless, despite their apparent similar fluid dynamics function, they differ substantially. The left ventricle (LV), that pumps oxygenated blood in the Aorta, is more powerful and presents a thicker myocardial muscle than the right ventricle (RV). This is because the LV must work against a high "afterload": the high pressure (80–120 mm Hg, in normal subjects) that is present in the initial part of the systemic circulation, whereas resistances along the pulmonary circulation are smaller and the afterload that the RV has to face during its pumping function is much lower (5–20 mm Hg). In summary, the two ventricles present the same global function in terms of flow generation, but RV flow is created against lighter level of pressure.

This study performs a parallel analysis of both RV and LV fluid dynamics and presents a comparative assessment of their global energetic and functional properties. The aim is to describe how the RV fluid dynamics differs from the LV, and verify the significance of the RV streamlined geometry in relation with the lighter function to which it is devoted. The underlying longer-term objective is to recognize global indicators of the normal RV fluid dynamics, building on top of the wider LV knowledge, to improve the ability of identifying alterations during the development of pathological conditions. A comparative understanding of RV and LV fluid dynamics can take further significance when the former is required to substitute an absent or hypoplastic LV in congenital defects.

## 2. Materials and Methods

### 2.1. Geometric Description

The geometric information for the cardiac structures were obtained by clinical imaging techniques, and the information were relative to healthy subjects made available for research in anonymous from partner institutions that ensured compliance with their local institutional ethical committee. The time-varying geometry of a normal RV has been extracted by 3D echocardiography; the moving borders are obtained by a semi-automatic procedure within a dedicated software (4DRVFunction, Tomtec Imaging Systems GmbH, Unterschleissheim, Germany) that also identifies the size and location of the tricuspid and pulmonary valves. The clinical image was recorded with 30 images per heartbeat with a voxel size of 1 mm, and the geometry was then provided with triangular elements with average mesh spacing about 4 mm. For this study, we evaluated the geometries of only one anonymous healthy case. The patient's ventricular clinical data are as follows: end-diastolic volume (EDV) = 106 mL, end-systolic volume (ESV) = 41 mL, stroke volume (SV) = 65 mL, ejection fraction (EF) = 61%, and the ratio of peak velocity blood flow from ventricular relaxation in early diastole to peak velocity flow in late diastole (E/A) = 1.7. These values are in line with results in literature for healthy case evaluated by different methods (Hudsmith et al., 2005; Bernard et al., 2017; van der Ven et al., 2020). The LV geometry has been obtained by the three long axis magnetic resonance imaging (MRI) acquisitions, from which the 3D shape below the valvular plane is reconstructed as previously described (Pedrizzetti et al., 2017); the temporal resolution was of 30 images per heartbeat and the average mesh spacing about 5 mm reconstructed from 12 transversal and 3 longitudinal slices. The positions of the mitral annulus and of the aortic outlet are also identified from the images.

**Figure 1:**
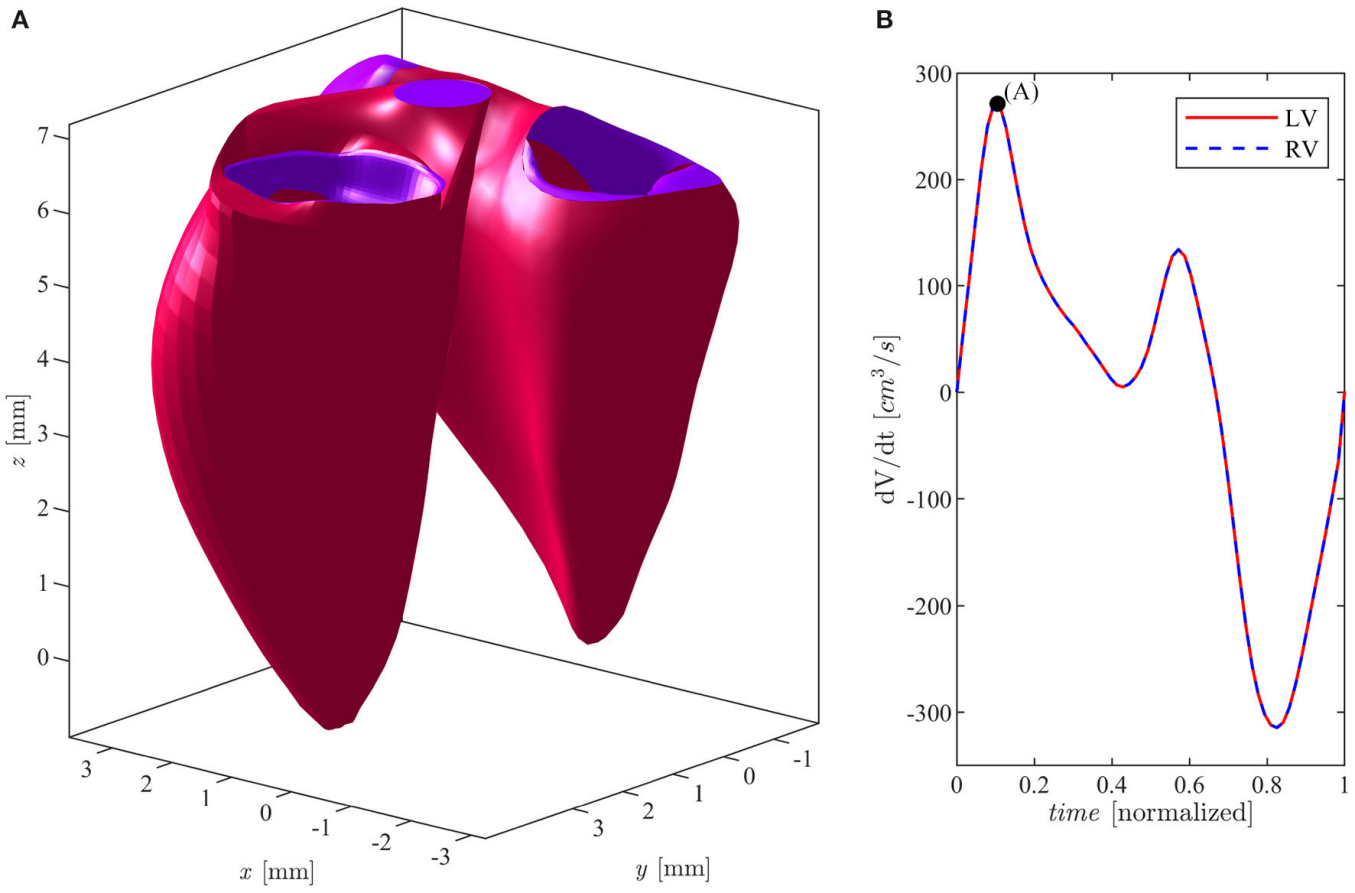
**(A)** Left and right ventricles in E-wave peak position. **(B)** Flow rate curve during cardiac cycle for both ventricles.

The volumetric time curves of both the LV and the RV in a same individual are very similar, they present close flow rates, and absolute volume values are sometimes slightly smaller in the RV. In order to avoid any confounding factor, we assumed identical volumetric time curves for both ventricles and the LV geometry is thus rescaled to this purpose as follows. First, a multiplicative expansion/contraction factor is applied to the minimum and maximum volumes to set the required values, and an intermediate factor, linearly changing from the minimum to the maximum, is applied to the intermediate values. Second, the rate of change is adjusted by a time modulation of the volume values during systolic contraction and during diastolic expansion. Similarly, the sizes of the valve at the RV inlet and outlet (tricuspid and pulmonary valves) are normally comparable to the corresponding ones in the LV (mitral and aortic valves). Therefore, the latter are assumed identical to those measured in the RV and the LV valvular plane is then completed with a bi-linear surface connecting the LV annulus with the valvular annuli as previously described (Collia et al., 2019a). In order to complete the validation of the clinical data, we compared the annulus diameters of both the mitral (MVd) and tricuspid (TVd) valves, as well as the aortic (AVd) and pulmonary (PVd) orifice, at the end of systole, with the existing literature (Capps et al., 2000; Dwivedi et al., 2014): MVd = TVd = 2.5 cm; AVd = PVd = 1.4 cm. These values fall within the physiological parameters of a healthy subject. The geometry of a normal mitral (bileaflet) valve, at the inlet of the LV, is obtained from computed tomography (CT) in the fully open (at peak diastole) and fully closed (during early systole) positions. The valve is then allowed to move between these two limiting positions as briefly described below. The two leaflets were allowed to move independently, each one associated with a degree of opening, say φ_1_(*t*) and φ_2_(*t*), for the anterior and posterior leaflets, respectively. The valve geometry is described by its coordinates **X**_*mv*_(ϑ, *s*, φ_1_, φ_2_), which represents a two-dimensional set of intermediate positions associated with the different degrees of leaflets openings (Collia et al., 2019b). This set of possible geometries is estimated by interpolation between the closed **X**_*mv*_(ϑ, *s*, 0, 0), and open ${\text{X}}_{mv}\left(\vartheta ,s,\frac{\pi }{2},\frac{\pi }{2}\right)$ configurations obtained from images as previously described. The geometry of a tricuspid valve, at the inlet of the RV, was described by a mathematical model in terms of the three degrees of freedom represented by the opening of each of the three leaflets. In this model, the three closed leaflets are assumed identical in a planar configuration, and each cusp opens along a circular trajectory and the geometry is built to connect the apex to the base with a quadratic profile ensuring continuous (horizontal) derivative. This valve surface is then accommodated to lay over the bi-linear surface matching the curved annulus. Following the same line used for the MV, the TV is described by its coordinates **X**_*tv*_(ϑ, *s*, φ_1_, φ_2_, φ_3_) where the three angles φ_1_(*t*), φ_2_(*t*), and φ_3_(*t*) correspond to the opening angle for the three leaflets, moving independently between 0 and $\frac{\pi }{2}$. The outlet valves, aortic for the LV and pulmonary for the RV, are described with a binary behavior, fully open or fully closed. The positioning of the valves in the ventricular geometry is specified by the ventricular recording itself and does not require explicit registration other than minor adaptation of the annulus. The numerical simulation is performed for each ventricle individually, as the two flows do not interact other than through the geometry. The relative position of the two ventricles is performed manually for visualization purpose and it is shown, with indication of valves, in Figure 1.

### 2.2. Fluid Dynamics

The numerical method was extensively described and validated in a dedicated methodological study (Collia et al., 2019a), where the valvular dynamics was compared with that obtained by a complete fluid–structure interaction (Meschini et al., 2018). In this section, we briefly recall the main points of the method used. The intraventricular fluid dynamics is evaluated by numerical solution of the Navier–Stokes and continuity equations

(1) $$\begin{array}{l} \frac{\partial v}{\partial t}+v\cdot \nabla v=-\nabla p+\nu \nabla^{2}v, \end{array}$$

(2) $$\begin{array}{l} \nabla \cdot v=0; \end{array}$$

where **v**(*t*, **x**) is the velocity vector field, *p*(*t*, **x**) is the kinematic pressure field, and ν is the kinematic viscosity (assumed 0.04*cm*^2^/*s*) of a Newtonian fluid. Solution is achieved by the immersed boundary method in a bi-periodic Cartesian domain as described in previous studies (Domenichini, 2008; Mangual et al., 2012, 2013; Domenichini and Pedrizzetti, 2016; Collia et al., 2019a). Time advancement is achieved using a fractional step method as follows. Velocity is preliminarily advanced in time by the Navier–Stokes (Equation 1) using a low-storage, third-order Runge–Kutta explicit scheme. This preliminary velocity, say $\hat{v}$, that does not satisfy the incompressibility constraint (2), is corrected by adding a potential field δ**v** = ∇*q*, such that **v** = $\hat{v}$+δ**v** satisfies the continuity and the boundary conditions. The correction potential is found by solution of the Poisson equation

(3) $$\begin{array}{l} \nabla^{2}q=-\nabla \cdot \hat{v}; \end{array}$$

and pressure is updated with *q* accordingly. Boundary conditions at the edge of the computational box are set periodic in the *x* and *y* directions, while they are zero pressure and normal velocity on the upper and lower ends along *z*, respectively. The 2D Fourier decomposition permits fast solution of the Poisson (Equation 3) as a sequence of tridiagonal systems for each harmonic. Boundary conditions are set on the moving immersed boundaries that comprise the ventricle geometry and valves surface. The boundary conditions are imposed on the intermediate velocity $\hat{v}$ at the end of the Runge–Kutta time advancement before imposing the correction obtained by Equation (3) (Domenichini, 2008).

Given that the immersed boundaries do not coincide with the computational grid, a local interpolation scheme is commonly used to transfer the precise boundary conditions at the surrounding computational points (Mittal et al., 2008; Meschini et al., 2018). In the present model, accounting that the physiological geometries present details not resolved in the imaging technology, that the used geometry represents only one realization within a range of natural variability, and that the boundary is mathematically represented as a surface of zero thickness, we have simplified the interpolation scheme by closing the cell containing the immersed boundary setting a speed equal to the average of the points falling into that cell. Furthermore, when calculating the Navier–Stokes equation at the closed cells corresponding to the soft tissues, the viscosity is artificially increased to its maximum stable value and thus avoiding unrealistic sharp edge boundaries and improving numerical convergence. This implication of this simplification was evaluated in a previous study for the LV (Collia et al., 2019a), and additional verification in the RV is reported in section 3.2.

The dynamics of the valvular leaflet is obtained by the constraint that the motion of the leaflet surface must match the velocity of the fluid at the position of the same surface. This is imposed in the least square sense over each individual leaflet, and gives rise to system of dynamic equation for the advancement of each leaflet opening angle. This procedure is described in details in Collia et al. (2019a). The result is a system of linear equations whose *i*th term reads

(4) $$\begin{array}{l} \left[\int_{A_{v}}\left(\frac{\partial {\text{X}}_{v}}{\partial \varphi_{i}}\cdot n\right)\left(\frac{\partial {\text{X}}_{v}}{\partial \varphi_{j}}\cdot n\right)dA\right]\frac{d\varphi_{j}}{dt}\\ \;=\int_{A_{v}}\left(v\cdot n\right)\left(\frac{\partial {\text{X}}_{v}}{\partial \varphi_{i}}\cdot n\right)dA; \end{array}$$

where ***n*** is the normal to the valvular surface and the subscript _*v*_ stands for either the mitral or the tricuspid valve (summation over *j* is implicit and extends to 2 or 3 for the mitral and tricuspid valve, respectively).

The aortic and pulmonary valves, which are downstream of the LV and RV flow fields, respectively, are modeled as a simple orifice with a surface that is open or closed. The aorta is considered open when the mitral valve is closed and the normal velocity, averaged over the position of the aortic valve surface, before setting boundary conditions, is directed outward. The same is true for the pulmonary valve, it is considered open when the tricuspid valve is closed, and the normal velocity is directed outward. In this way, it is not necessary to prescribe the open or closed state of the valve from global considerations because the exact start-to-end times of systole and diastole can be difficult to precisely define under pathological conditions. More details are given in Collia et al. (2019a).

### 2.3. Vorticity, Kinetic Energy, and Dissipation Rate

The formation of the vortex and its orientation within the ventricles influences the correct course of the flow throughout the cardiac cycle until its expulsion (Kilner et al., 2000; Pedrizzetti and Domenichini, 2005; Pedrizzetti et al., 2014). The computation of the average vorticity inside the ventricle is

(5) $$\begin{array}{l} \bar{\omega }=\frac{1}{V}\int_{V}|\omega |dV \end{array}$$

where *V*(*t*) is the ventricular volume, and ***ω***(*t*) = ∇ × **v** is the vorticity vector field.The kinetic energy (KE) of the blood is a fundamental component of the work done by the two ventricles, indicated as the movement of the blood within them (Prec et al., 1949; Carlsson et al., 2012; Garg et al., 2018) and is calculated as follows:

(6) $$\begin{array}{l} KE\left(t\right)=\frac{\rho }{2}\int_{V}v^{2}dV, \end{array}$$

where ρ is the blood density. The KE dissipation rate

(7) $$\begin{array}{l} D\left(t\right)=\rho \nu \int_{V}S_{ij}\frac{\partial v_{i}}{\partial x_{j}}dV, \end{array}$$

where *S* is the rate of deformation tensor and ν is the kinematic viscosity, provides a measure of the efficiency of blood flow (Hayashi et al., 2015) and it is an indicator of ventricular function (Seo and Mittal, 2013; Pedrizzetti and Domenichini, 2015). The total dissipation of KE during the heartbeat, Δ*KE*, is given by the time-integral of the dissipation rate (Equation (7)) during the entire heartbeat.

## 3. Verifications

### 3.1. Numerical Verification

The influence of numerical parameters on the computed flow properties was verified in numerous previous studies for the LV (Collia et al., 2019a). A grid refinement analysis is performed here for the RV in the present simulation conditions. We used a bi-periodic domain 10 × 6 × 12 cm that was previously verified to ensure absence of confinement effects, and report results for three different numerical resolutions ranging from a basic grid 128 × 96 × 192 points and 4, 096 time steps in one heartbeat, a more refined grid 192 × 128 × 256 points and 6, 144 time steps, and a further refined grid 256 × 192 × 320 points and 8, 192 time steps.

**Figure 2:**
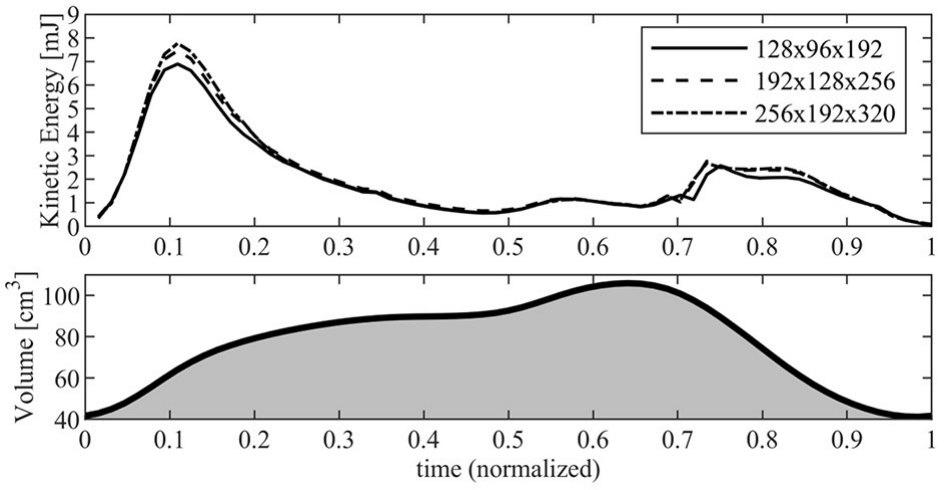
Kinetic energy inside the right ventricle (RV) evaluated with different numerical resolution. The continuous line corresponds to a grid made up of 128 × 96 × 192 points; dashed line corresponds to a grid made up of 192 × 128 × 256 points; dash-dot line corresponds to a grid made up of 256 × 192 × 320 points. The RV volume curve is reported below for reference.

Figure 2 reports the results in terms of KE contained inside the RV as computed with the three different resolutions. The KE shows a common behavior for the different resolutions, with the exception of the periods during diastolic peak (about *t* ≃ 0.11) and the early systolic acceleration (*t* ≃ 0.74) where the lowest resolution gives rise to a maximum underestimation of about 10% with respect to the values obtained with highest resolution, a difference that is significant for a global property. Differences are significantly reduced between the two more refined grids where a small disagreement remains only at peak diastole. These observations, with numerous others not shown for brevity, suggest that the most refined grid ensures numerical results that are close to numerical convergence. Based on this analysis, the present study used the most refined grid made of 256 × 192 × 320 points along the antero-posterior, left-right, and base-apex directions of the RV, respectively; the same resolution is employed for the LV simulations.

### 3.2. Immersed Boundary Model

**Figure 3:**
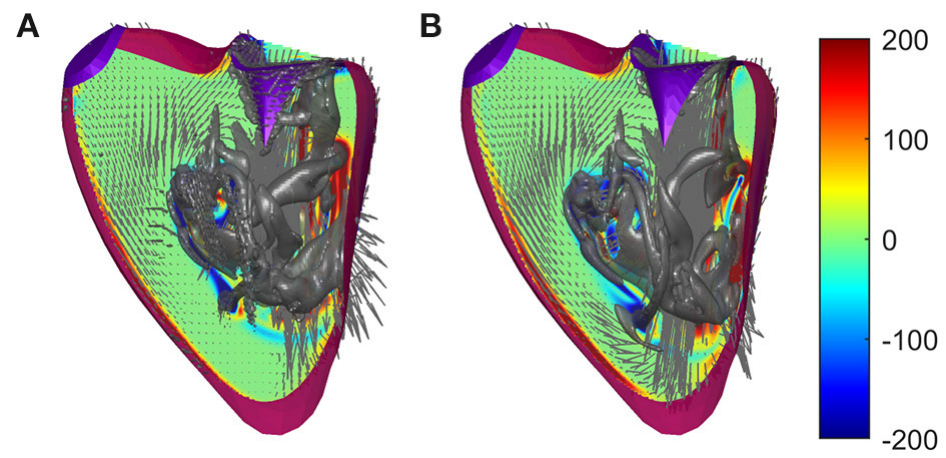
Flow fields in a normal right ventricle (RV) at peak E-wave computed **(A)** with regular viscosity and **(B)** with the enhanced viscosity at the immersed boundary using identical numerical parameters. The normal vorticity is shown by red to blue color from –200 to 200 units equal to the inverse of the heartbeat period and the velocity vector (ever 4 grid points) on a longitudinal plane; the three-dimensional gray surfaces represent the iso-surface of λ2 parameters.

The simplification that avoids unrealistic sharp edges described in 2.2 has already been evaluated and validated for the LV; in this section, it is also evaluated for the RV. The suitability of such a method has been suggested when the uncertainty in the position of the boundary is greater than the size of the grid because the method spreads the boundary condition on the computational cell. Here, we perform a comparative analysis between numerical solutions obtained with and without the introduction of such viscosity to verify the influence of this hypothesis with respect to the consideration of a smooth contour. The results show that the flow is largely unaffected by the introduction of this artificial limiting viscosity, with the only obvious difference being the presence of small flakes that are sometimes smoothed in the presence of the increased viscosity. For example, Figure 3 shows the flow fields at the peak of the E wave in the two cases.

The comparative analysis provides support by highlighting that this approach does not introduce non-physical phenomena other than the appearance of fluctuations in the size of the grid near the boundaries, a phenomenon that is really significant especially when the position of the boundary is not known precisely. Furthermore, this is useful in the presence of sharp boundaries that are the result of segmentation algorithms and the details of which may not be physically realistic, especially using the dipped boundary that can give rise to similarly unrealistic small scales in the fluid flow.

## 4. Results

### 4.1. Qualitative Vortex Flow Pattern

**Figure 4:**
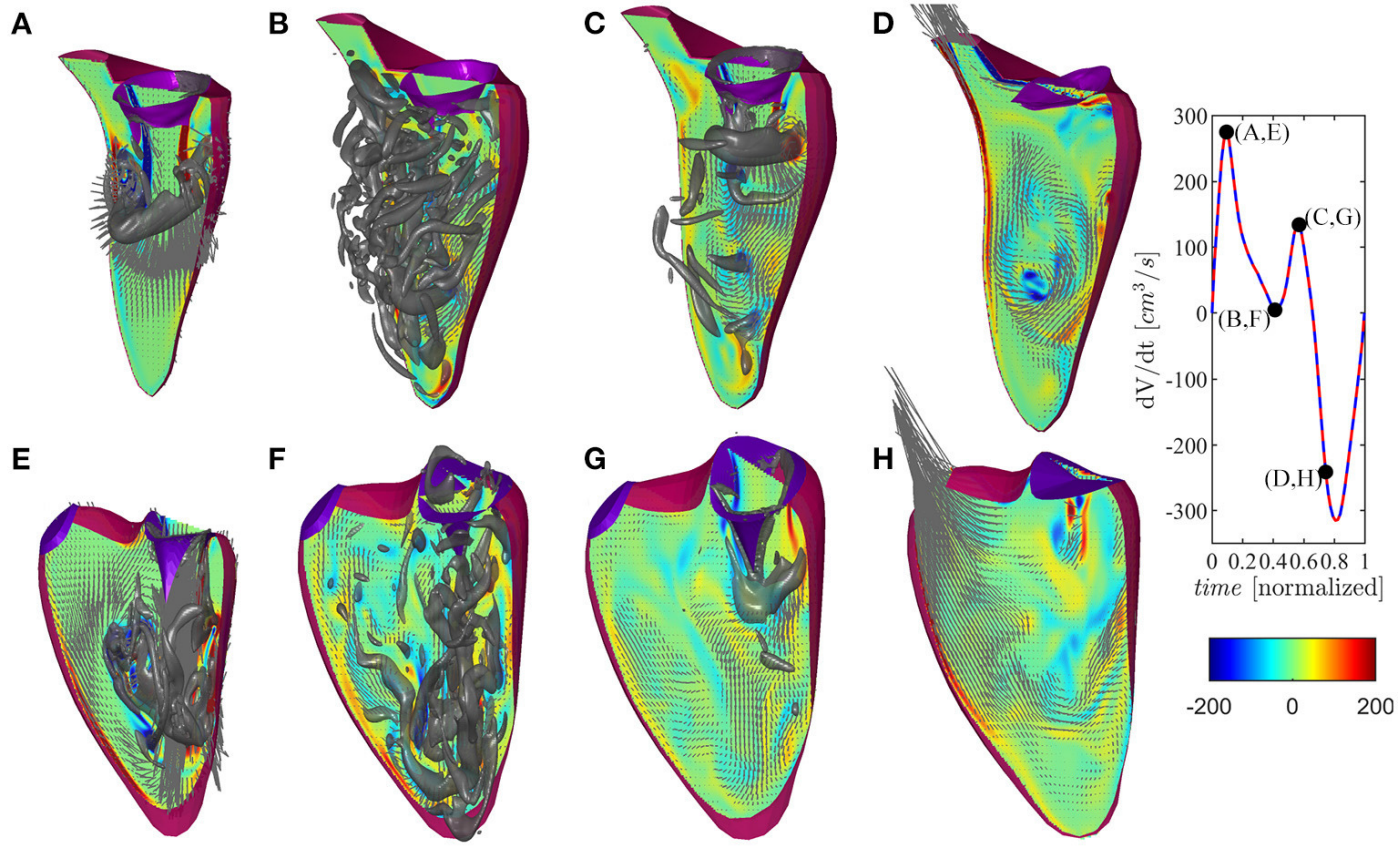
Flow pattern inside the left and right ventricles at four instants: **(A,E)** early filling wave, **(B,F)** diastasis, **(C,G)** atrial contraction wave, **(D,H)** beginning of systole, as indicated in the volume–rate curves in the insets [red color for left ventricle (LV) and blue color for right ventricle (RV)]. The normal vorticity is shown by red to blue color from -200 units to 200 units equal to the inverse of the heartbeat period and the velocity vector (ever 4 grid points) on a longitudinal plane; the three-dimensional gray surfaces represents the iso-surface of λ2 parameters.

The vortex flow pattern is depicted graphically in Figure 4 for the ventricles during (Figures 4A,E) first peak of diastolic inflow (E-wave), (Figures 4B,F) diastasis, (Figures 4C,G) second diastolic peak (A-wave), and (Figures 4D,H) beginning of systolic contraction (see Supplementary Movie 1 for the representation of both ventricles along the whole heartbeat).

The normal LV flow pattern is extensively described in literature (Pedrizzetti and Domenichini, 2015; Mittal et al., 2016; Khalafvand et al., 2017; Zhong et al., 2018). In brief, flow across the mitral valve enters as a jet inside the LV and develops a ring-like vortex structure below the aperture; the jet slightly deviates toward the nearby wall (postero-lateral side) because the vortex slows down on this side for image effect, and it then partly dissipates therein while interacting with the induced boundary layer. Therefore, the vorticity accumulates about the center of the chamber where a circulatory motion develops. This phenomenon, which can sometimes be accompanied by the development of weak turbulence, is further fed during the second diastolic filling wave. The intra-ventricular blood circulation ensures a good washout of LV apex and it supports the redirection of blood toward the LV outlet tract at the end of diastole, ready for systolic ejection. During the systolic phase, the flow has a well-ordered direction due to the swirling movement in the center of the ventricle that pushes the blood toward the aorta, thus starting the systole a moment earlier than the RV.

The flow field in the RV presents some qualitative differences with respect to the LV, which can be imputable to the different and peculiar RV geometry as previously described (Fredriksson et al., 2011; Mangual et al., 2012). The initial phase is similar to the LV, and it features the development of a ring-like vortex structure behind the tricuspid valve while the corresponding inflow jet approaches the apex of the shorter RV chamber. The vortex ring interacts with the close boundaries of this narrower chamber, particularly on the septal side that is flatter. Noticeably, during the entire diastole, the vorticity remains mainly confined in the region below the valve, dissipating therein without significant penetration into the slender region toward the outflow. During systolic contraction, the residual vorticity extends along the outflow tract adding a slightly helical pattern to the otherwise largely irrotational velocity field.

### 4.2. Vortex Formation and Energetic Analysis

The previous description is here analyzed in terms of synthetic parameters of physical relevance. The average amount of vorticity that is generated is initially comparable in the two ventricles because vorticity develops from the inlet jets that were taken with same annulus size and are crossed by the same volume rate. In both ventricles, the starting phase of the vortex formation process can be modeled as that of a vortex ring of radius *R*, comparable to that of the valvular annulus, and growing circulation Γ(*t*). In this model, the vortex circulation grows over time as $\frac{d\Gamma }{dt}≅\frac{1}{2}U^{2}$, for the roll-up of a vortex sheet, describing a velocity jump between the average velocity across the valve *U* and the lateral approximately quiescent fluid, that feeds the vortex at an average velocity approximately equal $\frac{1}{2}U$. The velocity *U* is given by the volumetric rate

(8) $$\begin{array}{l} U=\frac{1}{\pi R^{2}}\frac{dV}{dt}, \end{array}$$

where *V*(*t*) is the ventricular volume, and the average vorticity (Equation (5)) at the onset of diastolic filling can be estimated by

(9) $$\begin{array}{l} \bar{\omega }≅\frac{2\pi R}{V}\Gamma ≅\frac{2\pi R}{V}\int_{0}^{t}\frac{d\Gamma }{dt}dt≅\frac{1}{V}\frac{1}{\pi R^{3}}\int_{0}^{t}{\left(\frac{dV}{dt}\right)}^{2}dt. \end{array}$$

**Figure 5:**
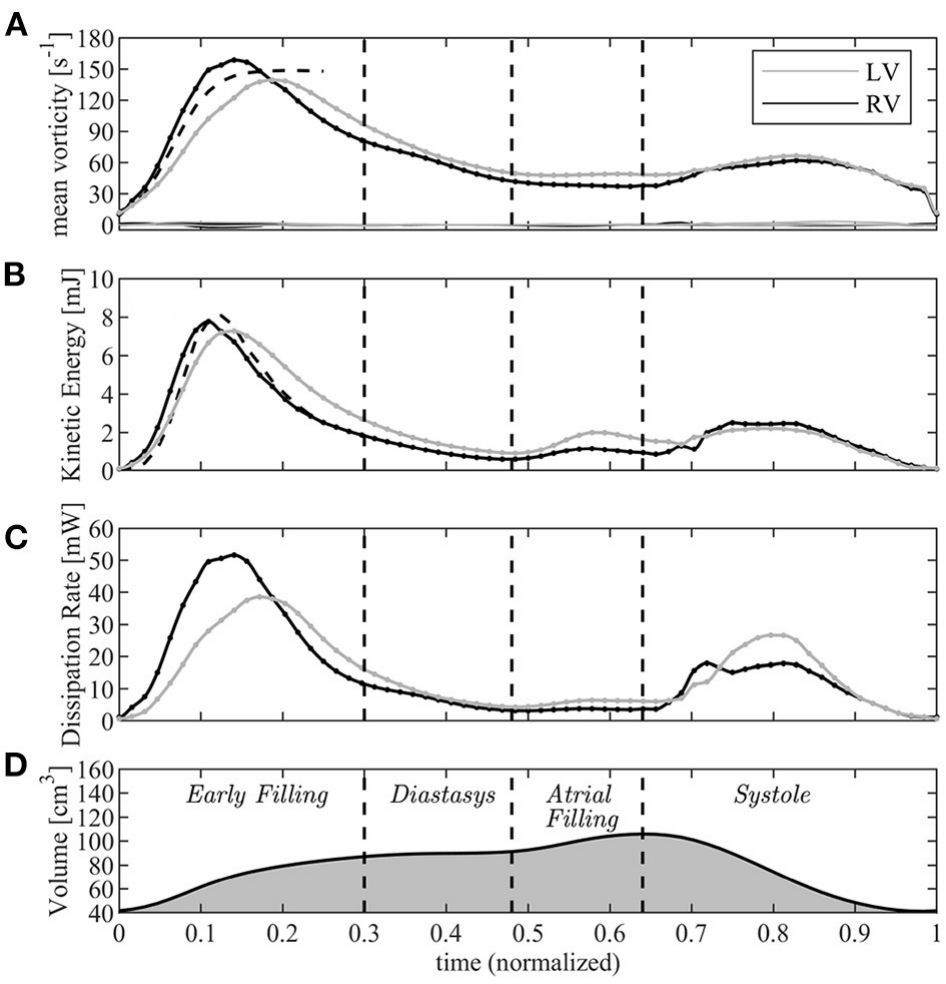
Graphical representation of the vorticity and energetic analysis in left ventricle (LV) (gray lines) and right ventricle (RV) (black); **(A)** mean vorticity, **(B)** kinetic energy, **(C)** dissipation rate, **(D)** volume curve (for timing reference). The dashed lines in **A** and **B** are the asymptotic short time estimates.

The time course of the average vorticity is shown in Figure 5A. The same figure reports the individual components of the vorticity very close to zero that should be zero up to numerical approximations to support the absence of nonphysical numerical effects. The initial asymptotic estimate (Equation (9)), overlapped with dashed line, confirms the picture of a comparable starting behavior for both ventricles. The slightly slower growth of the left ventricular vorticity at the onset of diastole is presumably due to the more irregular shape of the mitral orifice , where the vortex ring formation process is less comparable to that through a circular orifice.

After this phase, the fluid dynamics begin to differ between the left and right sides. While the average vorticity reduces in both ventricles for the natural vorticity dissipation phenomena, this process is more rapid in the RV where the vortex ring is constrained in a narrower chamber and interacts more rapidly with neighboring boundary layers. The transient nature of the tricuspid vortex ring and its quick dissipation was previously reported from clinical observations (Sengupta and Narula, 2013); vorticity decay is slower in the LV where it has room to maintain a weak circulation in the central regions of the wider chamber.

**Figure 6:**
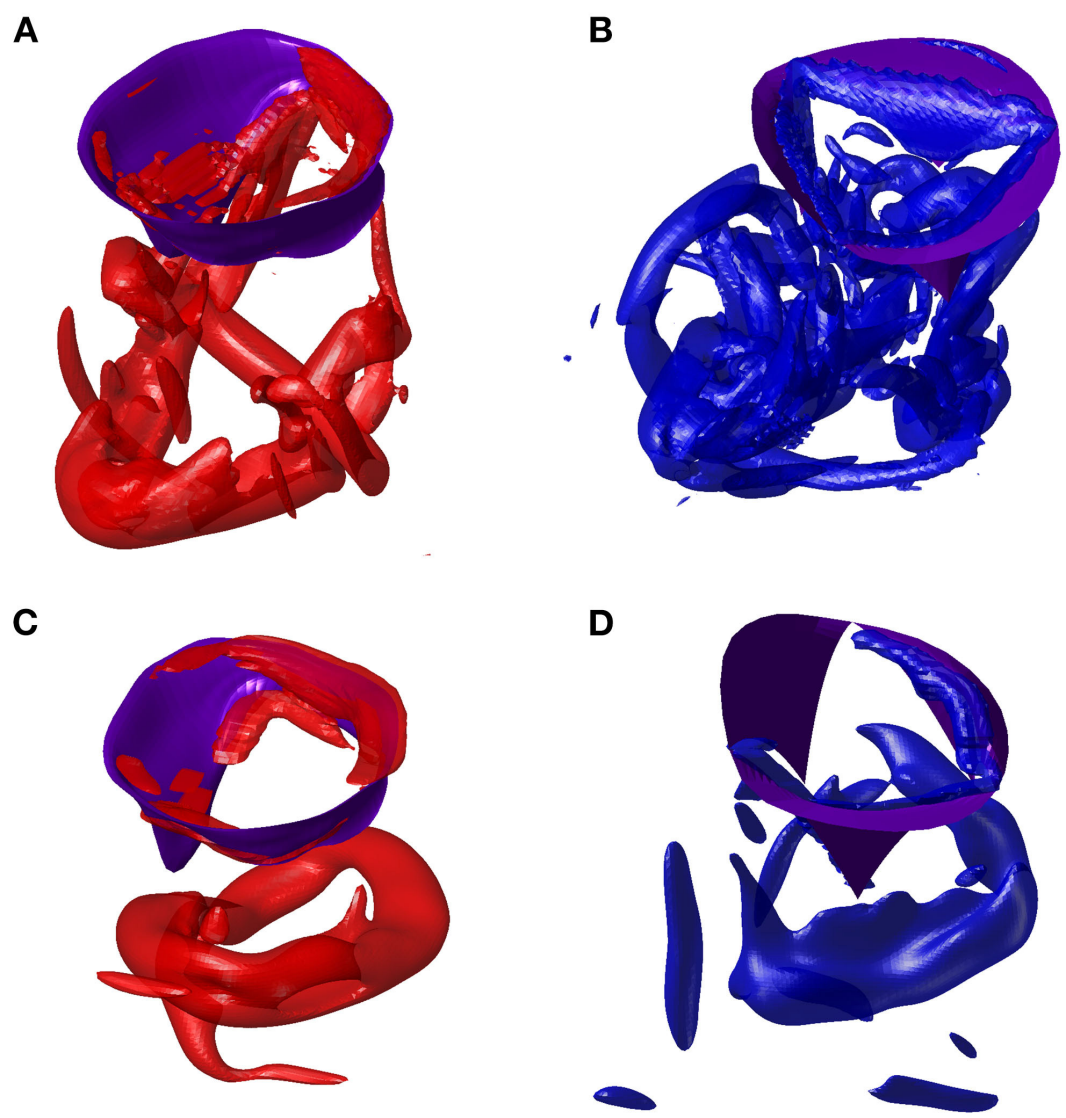
Vortex in E-wave peak of left ventricle (LV) **(A)** and right ventricle (RV) **(B)**. Vortex in A-wave peak of LV **(C)** and RV **(D)**.

At the end of diastasis, the coherent vorticity structure is mostly dissipated and the level of vorticity is mostly imputable to the region near the wall boundary. At the onset of atrial contraction, a second ring generates, however its strength is low and it is only able to provide a reduction in the decay and sustain the existing vorticity level. In support of this picture, the vortex structure is shown in Figure 6 for both ventricles during E- and A-waves. Vortex ring formation is similar in the two ventricles, as previously demonstrated *in vivo* with 3D phase-contrast magnetic resonance imaging (known as *4D Flow MR*) (ElBaz et al., 2014). At the time of atrial contraction, the coherence of the vortex structures is low in both ventricles, particularly in the RV.

This vorticity-based picture is analyzed in energetic terms in the following panels of the same picture (Figures 5B,C). The total KE (Equation (6)) is comparable for both ventricles in the initial phase when it is dominated by that of the entering jet that is similar in the two cases. Indeed, during the very early filling, the KE (Equation (6)) can be approximated by that of a cylindrical jet of velocity *U*, with cross-section equal to the annulus π*R*^2^ and length given by the time integral of velocity. This gives

(10) $$\begin{array}{l} KE≅\frac{1}{2}U^{2}\pi R^{2}\int_{0}^{t}Udt=\frac{V\left(t\right)-V\left(0\right)}{2\pi R^{2}}{\left(\frac{dV}{dt}\right)}^{2}; \end{array}$$

where the approximation (Equation 8) was used. This estimate, which is reported in Figure 5B with a dashed line, suggests that significant difference in KE between LV and RV can mainly be imputable due to difference in the dynamics of the valvular leaflets.

Approaching the diastolic peak, the RV is subjected to a larger dissipation of KE than the LV, a phenomenon that is correlated with the dynamics of vorticity confined in the narrower RV region below the valve whose absolute value decreases more rapidly. Therefore, after the first diastolic peak, KE remains some higher in the LV where it is partly conserved within the intraventricular circulatory pattern. The second diastolic wave in the LV can partly sum-up to the existing KE, whereas it enters in a more quiescent fluid in the RV.

At the beginning of systole, the RV starts its contraction in the basal inflow region while the contraction of the slender outflow tract begins after a small delay; this produces a light peristaltic effect at the onset of systole that facilitates the transfer of blood from the sub-valvular volume into the outflow region. It also quickly stretches and dissipates the vorticity still present in the basal flow, and progressively transforms into an irrotational motion while entering into systolic contraction. Differently, energy dissipation develops in the LV during the entire systole while the chamber-filling remaining vorticity stretches toward the Aorta. Results for KE can be compared to experimental observation performed *in vivo* with 4D Flow MR. These reported comparable diastolic values in the LV, while figures are about halved in the RV (Carlsson et al., 2012; Steding-Ehrenborg et al., 2016). The difference therein, however, is too large to be consistent with the estimate (Equation 8) and is must be imputable by effective differences between RV and LV volume rate and valvular size.

In the systolic phase, the systolic KE reaches values that are significantly smaller than those in diastole for both ventricles. This is justified by the fact that, during diastole, the high velocities of the inflow jet develop inside the ventricular volume and have a major role in the KE balance as shown by the approximation (Equation 10); differently, during systole, the ventricular KE only includes the lower values of the velocity field that converges toward the valve. The same 4DFlowMR studies reported a higher systolic KE wave; however, that balance included the high velocities in the proximal valvular region, we verified that the value rapidly changes when small portions of the outflow vessel are included in the balance.

### 4.3. Mechanical Work

The mechanical power (rate of change of mechanical work) is defined as the scalar product between force and velocity. In the case of a volume *V* of fluid with no volumetric forces, the only force is the pressure (ignoring viscous stresses) that acts normally to its bounding surface *S*. The power, *P*(*t*), of such volume of fluid is

(11) $$\begin{array}{l} P\left(t\right)=\int_{S}pn\cdot vdS=\int_{V}\nabla p\cdot vdV. \end{array}$$

where we used the Gauss theorem, and the fact that ∇·***v*** = 0 in an incompressible flow. The power (Equation (11)) is the hemodynamic power that is associated with blood flow moved by the pressure gradient, which is independent from the average value of pressure inside the ventricle, say *p*_0_(*t*). The power (Equation (11)), as shown in Pedrizzetti et al. (2021), represents an additional terms related to blood flow and does not include the work performed to achieve a change of volume against a pressure. That work is globally zero in an insulated system filled with incompressible fluid, because the work is performed by the ventricular contraction in the presence of a pressure, which is commonly expressed as

(12) $$\begin{array}{l} W_{0}\left(t\right)=\int p_{0}dV, \end{array}$$

is equal and opposite sign of the work produced by ejection of the same volume of fluid at the same pressure (and vice versa during ventricular expansion).

**Figure 7:**
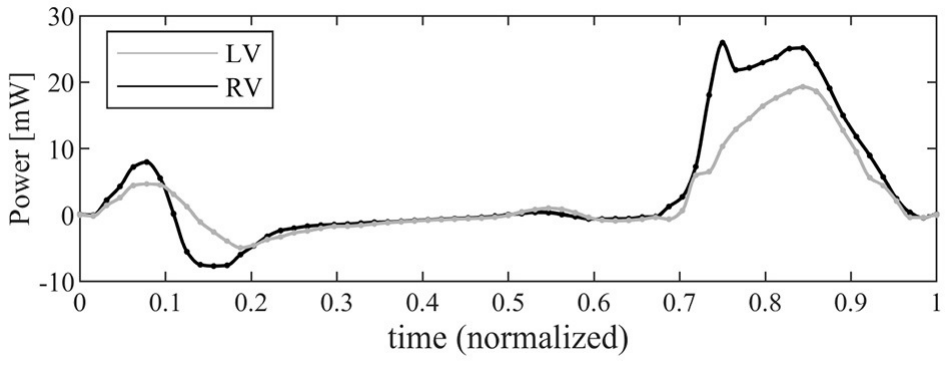
Graphical representation of the instantaneous power associated with blood flow for left ventricle (LV) (gray line) and right ventricle (RV) (black).

The time course of the flow power (Equation 11) is drawn in Figure 7 for both ventricles. The profile in systole shows that, for a same stroke volume, the RV transfer more power *P* to the blood than the LV does. This is due to its streamlined geometry where a contraction is efficiently transformed in blood flowing toward the RV outflow tracks; differently, the LV has to redirect upward the weakly turbulent motion originally directed downward, encountering a resistance that is only partially mitigated by the LV circulatory pattern. In both ventricles, the power during diastole is much smaller; here the differences are associated with the different valvular geometries that ensure a more vigorous early inflow in the RV followed by a sharper deceleration.

The total systolic work, *W*, obtained by integrating the power curve during systole, is reported in Table 1 for both the RV and the LV. The additional work, *W*_0_, required to work against the afterload depends on the effective time profile of the pressure in the pulmonary artery or the aorta, respectively. An estimation, computed with Equation (12), is reported in Table 1 just for the sake of comparison assuming an average value of afterload *p*_0__*RV*_ ≈ 10*mmHg* and *p*_0__*LV*_ ≈ 100*mmHg*. These figures show that most of the LV work is made against pressure in aorta and its efficiency in terms of flow is negligible in a global balance. Differently, the RV produces more mechanical work than the LV with the same stroke volume. The work made by the RV for developing flow is just one order of magnitude lower in comparison to that needed to overcome the afterload; therefore, this may rapidly play a role even in the global balance in the presence of pathological or stress conditions.

**Table 1:** Systolic work, *W*, associated with blood flow and that, *W*_0_, required to overcome afterload (estimated).

	***W*[*mJ*]**	***W*_0_[*mJ*]**
RV	4.8	≈ 86
LV	3.5	≈ 860

## 5. Discussion

Recent literature recognized the diagnostic and prognostic significance of LV fluid dynamics while such understanding did not progress at a comparable pace for the RV (Kheradvar et al., 2019). The reason is the presumed lower clinical relevance of the RV, in addition to its complex geometry that is elusive to imaging methods. However, it is now well recognized that the RV plays a central role is several cardiac dysfunctions (Tretter and Redington, 2018); it is altogether evident that results about LV fluid mechanics cannot be directly transferred to the RV. This study provided an initial comparative analysis between LV and RV fluid dynamics.

The early diastolic flow presents similar features in both RV and LV, dominated by the vortex formation process. After that, RV flow features a higher dissipative state, whereas the LV preserves an amount of circulation and KE during the whole diastole. At the onset of systole, the RV streamlined geometry efficiently redirects blood toward the outflow.

It is demonstrated that most of the LV mechanical work is made against pressure in aorta and its efficiency in terms of flow is negligible in a global balance. This does not imply, however, that the presence of flow disturbance cannot have a role in the LV, as they can still create regional imbalances, pressure fluctuations, that may stimulate adaptive feedback despite they do not alter global energetic balances (Pedrizzetti et al., 2014). Indeed, the LV circular shape is properly designed to withstand high pressure values scarifying the efficiency of flow generation.

Differently, the RV behaves as a reservoir during the diastolic phase where the incoming fluid is accommodated in the anterior volume dissipating the incoming energy; therefore, minor changes in regional stiffness can deviate the filling process and alter the dynamical interaction with the surrounding tissue. At the onset of systole, the RV features an efficient flow conduction that, for a same stroke volume, generates more propulsive power than the LVs, an efficiency that can be modified by regional contractile adaptations in either entity or timing. This analysis supports the suggested varying nature of RV function during the phases of the cardiac cycle (Sengupta and Narula, 2013). Importantly, the work made by the RV for developing flow is also non-negligible with respect to that needed to overcome the low blood pressure of the pulmonary arteries; therefore, small loss of efficiency in RV fluid dynamics can impact on energetic balance and play a role in the progression of pathology (Noordegraaf et al., 2017).

The present analysis did not include some phenomena that may further differentiate the flow efficiency in the two ventricles. One is the different RV conduction system that produces a slightly differentiated contraction pattern. Other factors, like the role of respiration on venous return, that supports right atrial filling, or the continuous coronary flow in the RV that ensures a high perfusion over the thin wall during the whole cycle.

## Data Availability Statement

The raw data supporting the conclusions of this article will be made available by the authors, without undue reservation.

## Ethics Statement

Ethical review and approval was not required for the study on human participants in accordance with the local legislation and institutional requirements. Written informed consent for participation was not required for this study in accordance with the national legislation and the institutional requirements.

## Author Contributions

All authors listed have made a substantial, direct and intellectual contribution to the work, and approved it for publication.

## Conflict of Interest

The authors declare that the research was conducted in the absence of any commercial or financial relationships that could be construed as a potential conflict of interest.
