# Supplementary material for: Comparative Analysis of Right Ventricle Fluid Dynamics
Source: Front Bioeng Biotechnol. 2021 Jul 6;9:667408. doi: 10.3389/fbioe.2021.667408 (PMC8290199; doi:10.3389/fbioe.2021.667408)
Supplement: Supplementary file 1 [file Data_Sheet_1.PDF]

Supplementary Material

1 SUPPLEMENTARY DATA

Movie 1. Flow pattern inside the left and right ventricles during the entire heartbeat. The three dimensional surfaces (red color for LV and blue color for RV) represents the iso-surfaces of $\lambda 2$ parameters.
